# Supplementary material for: Using information theory to optimise epidemic models for real-time prediction and estimation
Source: PLoS Comput Biol. 2020 Jul 1;16(7):e1007990. doi: 10.1371/journal.pcbi.1007990 (PMC7360089; doi:10.1371/journal.pcbi.1007990)
Supplement: S1 Text — (PDF) [file pcbi.1007990.s001.pdf]

Using information theory to optimise epidemic models for real-time prediction and estimation

Kris V Parag[*, 1] and Christl A Donnelly[1, 2]

[1]MRC Centre for Global Infectious Disease Analysis, Imperial College London, London, W2 1PG, UK
[2]Department of Statistics, University of Oxford, Oxford, OX1 3LB, UK
[*]Email: k.parag@imperial.ac.uk

S1 Text

Epidemic renewal models

Consider an incidence curve over times $1 \leq s \leq t$ for an epidemic with effective reproduction number and total infectiousness at t of R_t and Λ_t. While R_t describes the branching of the epidemic (the number of secondary cases originating from a primary one), $\Lambda_t := \sum_{s=1}^{t-1} I_{t-s} w_s$, controls how past infected cases propagate new infections, via the generation time distribution, which is defined by w_s. Here w_s is the probability that a primary case takes between $s-1$ and s days to generate a secondary case [1]. This distribution is intrinsic to a disease and $\sum_{s=1}^{b} w_s = 1$ for some memory time b.

We make the common assumptions that the generation time distribution is known and does not change with time [2]. The quantities R_t and Λ_t completely describe the transmissibility of an epidemic – an idea formalised by the renewal model [1]. The renewal model discretises the fundamental Euler-Lotka reproduction equation from ecology and evolution and states that $I_t \sim \text{Poiss}(R_t \Lambda_t)$ [3]. This relationship is applicable to any population biology problem where observed sample counts are used to estimate underlying growth rates.

Generally R_t is unknown and must be inferred from (I_1^t, Λ_1^t). Its log-likelihood under the standard renewal model, $l_t^{(1)} = \log \mathbb{P}(I_t, \Lambda_t \mid R_t)$ is [4]

$$l_t^{(1)} = I_t \log R_t - R_t \Lambda_t + \zeta_t, \tag{S1}$$

with $\zeta_t = -\log I_t! + I_t \log \Lambda_t$ collecting terms that do not depend on parameter R_t. The superscript of $l_t^{(1)}$ highlights that this model employs a unit window length, and hence only uses (I_t, Λ_t) to infer R_t. While this construction maximises model flexibility, R_t estimates can be noisy and over-fitting is likely [5].

Grouping is therefore employed. This assumes that the reproduction number, denoted $R_{\tau(t)}$, is constant over the past k time units and leads to a

piecewise-constant function that classifies between meaningful and negligible reproduction number changes [2]. The grouped log-likelihood function, $l_t^{(k)} = \log \mathbb{P}(I_{t-k+1}^t, \Lambda_{t-k+1}^t \mid R_{\tau(t)})$, with parameter-independent term $\zeta_{\tau(t)} = \sum_{s\in\tau(t)} - \log I_s! + I_s \log \Lambda_s$ can be composed as

$$l_t^{(k)} = i_{\tau(t)} \log R_{\tau(t)} - R_{\tau(t)}\lambda_{\tau(t)} + \zeta_{\tau(t)}, \tag{S2}$$

with grouped sums $i_{\tau(t)} := \sum_{s\in\tau(t)} I_s$ and $\lambda_{\tau(t)} := \sum_{s\in\tau(t)} \Lambda_s$. At $k = 1$ we recover Eq. (S1) from Eq. (S2).

The maximum likelihood estimates (MLEs) and Fisher information (FI) of Eq. (S2) provide insight into the benefits of k-grouping. The MLE facilitates unbiased inference, while the FI bounds the uncertainty around the MLE (it measures the inverse of estimate variance) [6]. The MLE, $\tilde{R}_{\tau(t)}$, is the solution to ${\partial l_t^{(k)}}/{\partial R_{\tau(t)}} = 0$, while the FI is $\mathbb{E}[-{\partial^2 l_t^{(k)}}/{\partial R_{\tau(t)}^2}]$ [6]. We actually compute the FI for the square root of $R_{\tau(t)}$, $\mathcal{I}(2\sqrt{R_{\tau(t)}})$, as it is known to have optimal properties [7]. The MLE and FI can then be derived as [5]

$$\tilde{R}_\tau = i_{\tau(t)}\lambda_{\tau(t)}^{-1} \qquad \text{and} \qquad \mathcal{I}(2\sqrt{R_{\tau(t)}}) = \lambda_{\tau(t)}. \tag{S3}$$

Comparing Eq. (S3) to equivalent expressions at $k = 1$ reveals the impact of grouping. We find that $\tilde{R}_{\tau(t)} = \sum_{s\in\tau(t)} ({\Lambda_s}/{\lambda_{\tau(t)}})\, \tilde{R}_s$ and $\mathcal{I}(2\sqrt{R_{\tau(t)}}) = \sum_{s\in\tau(t)} \mathcal{I}(2\sqrt{R_s})$. The grouped MLE is hence a weighted moving average of the ungrouped MLEs, explaining why noise is reduced. The grouped FI is a linear summation of ungrouped FIs, implying that estimate precision also increases with grouping. Unfortunately, these advantages come at the expense of elevated tracking bias. At the extreme of $k = t$, for example, $\tilde{R}_{\tau(t)}$ is a stable t-point average that can only be gradually perturbed by new incidence data. Thus, we trade the sensitivity to rapid R_s changes for smaller estimate variances. The need to formally mediate this trade led us to adapt the APE metric.

Prospective model selection

In [7] an approximate minimum description length (MDL) solution was proposed for retrospectively selecting a different but related k defining the non-overlapping window size optimising historical reproduction number estimates. This method, by exploiting an often neglected aspect of model complexity, known as parametric complexity [8], was shown to outperform standard measures such as Akaike (AIC) and Bayesian information criteria (BIC). While this method is not applicable here, as prospective performance requires different optimisations [9], we heed the lesson about accounting for parametric complexity.

The criterion we propose is the APE [10], which also approximates the MDL, but with an emphasis on prediction. The APE values models on their ability to generalise i.e. predict unseen data from the generating process [11]. Practically, this is implemented by sequentially predicting the data observed at time $s + 1$ (i.e. one-step-ahead of s) using the subset of data preceding it [9]. This means that we causally predict I_{s+1} at every s given a k-window back in time of

$\tau(s) = \{s, s-1, \ldots, s-k+1\}$. This window is truncated if $s < k$ so that $\min \tau(s) \geq 1$. We then evaluate our prediction (e.g. the posterior mean $\hat{I}_{s+1}$) against the observed I_{s+1}.

The k minimising the cumulative one-step-ahead prediction error up to the present t, which we term k^*, gives the renewal model that best predicts the unseen datum at $t+1$. We generally do not use $\hat{I}_{s+1}$ directly, but instead obtain its full predictive distribution $\mathbb{P}(x \mid I_{s-k+1}^{s})$, with x as some value of the predicted incidence at time $s+1$. The APE is defined as a cumulative log-score

$$\mathrm{APE}_k = \sum_{s=1}^{t-1} - \log \mathbb{P}\left(I_{s+1} \mid I_{s-k+1}^{s}\right). \tag{S4}$$

The optimal window, $k^* := \arg\min_k \mathrm{APE}_k$, is easy to compute provided the predictive distribution in Eq. (S4) is calculable. Fig 1 of the main text illustrates the APE approach. Eq. (S4) is general and applies to any statistical model for which one-step-ahead predictions can be obtained with I_s representing some type of data from which a time-varying parameter R_s is to be inferred.

By using the complete posterior predictive distribution the APE appropriately accounts for predictive uncertainty and is specialised to the problem of interest. A point-estimate alternative to APE, known as predictive mean squared error (PMSE), can be used when this distribution is not available [10] and is defined as $\mathrm{PMSE}_k = {}^1/_{t-1} \sum_{s=1}^{t-1} (I_{s+1} - \hat{I}_{s+1})^2$. While the PMSE might not be as tailored to the problem of interest, it can be easier to compute and both metrics converge when errors are normally distributed [11]. Other score functions can also be used when application-specific insights are available [12].

The APE metric has formal links to Bayesian model selection (BMS). BMS also includes parametric complexity and is asymptotically equivalent to the MDL when Jeffreys prior is used within the BMS [8, 13]. Interestingly, because any joint distribution can be decomposed as $-\log \mathbb{P}\left(I_1^t\right) = \sum_{s=1}^{t-1} - \log \mathbb{P}\left(I_{s+1} \mid I_1^s\right)$ $= \sum_{s=1}^{t-1} - \log \mathbb{P}\left(I_{s+1} \mid I_{s-k+1}^{s}\right)$, APE, under certain regularity conditions, is equivalent to both BMS and other MDL approximations (such as the one in [5]). For more details see [14] and [8]. The latter equality is from the finite memory of the renewal model, which depicts the non-stationary nature of epidemics. We assume $-\log \mathbb{P}(I_1) = 0$ in this decomposition as an initial condition.

However, the APE is simpler and more transparent, requiring no difficult integral evaluations [5]. Consequently, the APE not only accounts for parametric complexity (implicitly), but also applies to models of arbitrary complexity [11]. The drawbacks of APE are that it requires the data to be ordered in time, and being data-driven, its computational complexity increases linearly in both the number of models to be assessed and the size of t [15]. Overall, APE provides a simple and optimal solution to window selection, which surprisingly has not penetrated the epidemiological or ecological literature.

Acknowledgments

KVP and CAD acknowledge joint Centre funding from the UK Medical Research Council and Department for International Development under grant reference MR/R015600/1. CAD thanks the UK National Institute for Health Research Health Protection Research Unit (NIHR HPRU) in Modelling Methodology at Imperial College London in partnership with Public Health England (PHE) for funding (grant HPRU-2012–10080).

References

1. C Fraser. Estimating individual and household reproduction numbers in an emerging epidemic. *PLOS One*. 2007;8:e758.

2. A Cori, N Ferguson, C Fraser and S Cauchemez. A new framework and software to estimate time-varying reproduction numbers during epidemics. *Am. J. Epidemiol.* 2013;178(9):1505–12.

3. J Wallinga and M Lipsitch. How generation intervals shape the relationship between growth rates and reproductive numbers. *Proc. R. Soc. B.* 2007;274:599–604.

4. C Fraser, D Cummings, D Klinkenberg, D Burke and N Ferguson. Influenza transmission in households during the 1918 pandemic. *Am. J. Epidemiol.* 2011;174(5):505–14.

5. K Parag and C Donnelly. Adaptive estimation for epidemic renewal and phylogenetic skyline models. *Syst. Biol.* 2020;syaa035.

6. E Lehmann and G Casella. *Theory of Point Estimation*. Springer-Verlag, second edition; 1998.

7. K Parag and O Pybus. Robust design for coalescent model inference. *Syst. Biol.* 2019;68(5): 730–43.

8. P Grunwald. *The Minimum Description Length Principle*. The MIT Press; 2007.

9. P Dawid. Present position and potential developments: Some personal views. Statistical theory. The prequential approach. *J. R. Stat. Soc A,*. 1984;147:278–92.

10. J Rissanen. Order estimation by accumulated prediction errors. *J. Appl. Prob.* 1986;23:55–61.

11. E Wagenmakers, P Grunwald, and M Steyvers. Accumulative prediction error and the selection of time series models. *J. Math. Psychol.* 2006;50:149–166.

12. P Dawid. Prequential data analysis. *In Current Issues in Statistical Inference: Essays in Honor of D. Basu*. 1992;113–26.

13. J Rissanen. Fisher information and stochastic complexity. *IEEE Trans. Info. Theo.* 1996;42(1): 40–7.

14. J Myung, D Navarro and M Pitt. Model selection by normalized maximum likelihood. *J. Math. Psychol.* 2006;50:167–9.

15. M Hansen and B Yu. Model selection and the principle of minimum description length. *J. Amer. Stat. Assoc.* 2001;96(454):746–74.
