# Supplementary material for: Using information theory to optimise epidemic models for real-time prediction and estimation
Source: PLoS Comput Biol. 2020 Jul 1;16(7):e1007990. doi: 10.1371/journal.pcbi.1007990 (PMC7360089; doi:10.1371/journal.pcbi.1007990)
Supplement: Attachment — Submitted filename: plos_renewal_review.docx [file pcbi.1007990.s002.docx]

Renewal equation-based methods are frequently used to estimate the reproductive number ($R$) during infectious disease outbreaks. During an outbreak the $R$ varies with time, for instance due to susceptible depletion. Therefore, one challenge (and also use) for renewal-equation approaches is to estimate this variation. As the authors note, estimating variation in $R$ is typically accomplished using moving-window methods: dragging a window of a fixed size $k$ across the time series and estimating $R$ using data that fall within the window, assuming that $R$ is approximately constant within the window.

As a moving-window based method, this then poses the perennial problem of how to choose the window size $k$. To date, in the epidemiological literature this decision has typically been made on heuristic grounds. The contribution of this paper is to present a much-needed systematic way of making this decision, using techniques from information theory.

I consider the novel methodology presented a useful contribution to the disease modelling literature, and that the paper will ultimately be suitable for publication in PLoS Computational Biology.

In the main, the methodology is explained clearly, in particular in the text, however I have few suggested comments that I feel would improve the paper and aid the reader in using the techniques presented. I do not think any of my suggestions will require the authors to perform any new analyses or generate any new data.

1) As the authors state in the introduction, they intend their method to be integrable “within existing real-time frameworks”. When reading the methods and the first part of the results (Figures 2-3) I was left unsure whether this was possible. The text gave me the impression that it was necessary to perform the analysis on the entire time series in order to use APE to find $k^{*}$. This would obviously make the method useless in an unfolding outbreak situation, where a decision would need to made about choices of $k$ early on.

The authors later indirectly address this point in the section on “Diagnosing Efficacy of Control” and in Figure 4, which for me is actually the most important figure and section in the paper. I think it’s present framing in terms of control efficacy might obscure/undersell these results and it’s connection with the possibility for “integration within existing real-time frameworks”. I suggest the authors rework this section and expand the discussion of prospects for real-time implementation based on their findings. A few clarifying comments in the methods about how APE could be used in real-time would also help address this.

2) For most of the examples the APE appears to select a minimal/maximal $k^{*}$: either 2 or $t/2$. Is this a general result, are there examples of the selection of intermediate values? It it illuminating to plot the APE curve as a function of $k$? e.g. is it a strictly monotonic function or does it possess bimodalities? This information is sort of contained in Figure 5 through plotting multiple lines for different values of $k$, but it is a little hard to interpret. Is $\text{APE}(k)$ flat at the change-times? Some explanation of these observations seems essential to me. For instance one take-away from this paper for me is that, even if they don’t use APE, users of renewal-equation methods (e.g. EpiEstim) would be advised to pick both the minimal and maximal values of $k$, as one of them will be the best.
3) Figure 1 is very dense and I found challenging to follow the algorithm. For instance, I took away from the figure that $k_{1}$ and $k_{2}$ were calculated at different time points ($t_{1}$ and $t_{2}$), using different data — whereas they are calculated using moving windows. I wonder if an annotated simulated example with multiple panels (e.g. simulated time series in panel a, panel b and c shows estimates of $R$ using $k_{1}$ and $k_{2}$ respectively, then the APE algorithm in panel d) might frame the problem and illustrate the algorithm better.
4) Due to the number of different simulated scenarios analysed in the paper (6), it is difficult to comprehend Figure 2 and extract the key pieces of information. I also found the cyan confidence intervals a little hard to make out on a white background and some of the axis labels too small to read without zooming in. Is it possible to relegate some of the examples to the supplemental material and focus on one or two important ones in Figure 2?

Minor comments:

5) At the start of the methods it states “incidence or number of infected cases in an epidemic at present time t”. The wording of this is slightly ambiguous, as “number of infected cases in an epidemic at present time t” suggests the total number of infectious individuals present in the population rather than the number of new cases (i.e. incidence) studied in the paper.
1) Please add left and right y-axis labels to Figure 3 and 6.
2) Please add left y-axis labels to Figure 5.
