# Supplementary material for: Using information theory to optimise epidemic models for real-time prediction and estimation
Source: PLoS Comput Biol. 2020 Jul 1;16(7):e1007990. doi: 10.1371/journal.pcbi.1007990 (PMC7360089; doi:10.1371/journal.pcbi.1007990)
Supplement: Attachment — Submitted filename: plos_revisions.docx [file pcbi.1007990.s003.docx]

**Responses to Reviewers: PCOMPBIOL-D-20-00012**

We thank the reviewers and editors for their constructive and insightful comments. Our responses to each point, as well as our corresponding edits to the main text, are listed below. We include both clean and tracked-changes versions of the revised text.

**Associate Editor**

I congratulate the authors on a very nice manuscript that was well received by the reviewers. Both reviewers were complimentary of the methods, though they raised a number of concerns about the presentation. I would encourage the authors to revisit the figures, as both reviewers found that they were challenging to interpret.

Thanks, figures have now been reworked – see responses below.

Specifically:

1. Please increase the font size for figure labelling

Done – now at size 18, was at 14.5.

2. If possible, consider limiting the number of scenarios presented in the multi-panel figures (particularly Fig 2). This could be reduced to a smaller number of exemplary scenarios (e.g. a,f,c,e where the latter two reflect scenarios exemplary of interventions)

Fig 2 has now simplified into a new Fig 2 and Fig 3, containing two panels each.

3. Figure 1 is quite dense, and I agree with R2 that breaking this up into panels that could be labelled, and this referenced in the legend, would help with interpretation.

Figure 1 has now been reworked to a composite-panelled form with improved annotations. Hopefully the flow of ideas and the algorithm is now clear.

4. Consider using a color palettes that have greater contrasts.

The cyan lines/shading have been replaced with a clearer blue.

Both reviewers comment on the tendency for k* to be estimated at the minimal (both reviewers) or maximal (R2) values. Please elaborate on this phenomenon.

This is not a general property of the APE approach. The simulated examples in the new Fig 2 and Fig 3 show that a range of intermediate window lengths were chosen with k* = {30, 9, 7, 13} from a range of 2 to about 97/98. However, when rapid changes in transmissibility occur (as in the new Fig 5 and Fig 6) it is more likely for k* to be estimated at its minimal value due to the strong non-stationary behaviour exhibited by these epidemics. If APE were applied either side of these rapid changes and hence did not need to account for them then it would also settle on intermediate values where justified. See my later responses.

Please consider the suggestion 1 of R2. If it is possible to clarify the applicability in real-time, I suspect that would appeal to readers.

Additional text has now been included emphasising how the APE algorithm can be used to provide real-time insight into an ongoing epidemic. See my later responses.

Please address the minor comments of R2 and comments 1&2 of R1.

Done, see below.

**Reviewer: 1**

This was an interesting paper that adds to the suite of methods for analysing case report data. In particular it provides a useful measure of the “window size” over which the reproductive ratio Rt should be calculated. As such, this could prove to be a powerful tool. On the whole I found the paper to be extremely well written, and I only have a few minor comments.

Many thanks for the positive appraisal.

1. In the abstract, I find the phrase “most succinctly describes” to be rather vague and confusing, could the authors seek a more informative description?

This sentence has now been reworked as ‘…which deems the model capable of describing the observed data using the fewest bits as most justified.’

2. Page 9, the choice of a Gamma distribution for the posterior should be better motivated.

This choice is mostly in keeping with existing formulations of the renewal model for infectious disease epidemics. Specifically, in Cori et al 2013 and in Cauchemez et al 2006 a gamma prior is placed on R_t_ which results in a gamma posterior by conjugacy. We have adopted and extended this framework. The choice of a gamma prior is useful since gamma distributions can fit many unimodal distributions on the positive real line and under a mean and log-mean constraint have the maximum entropy property. Some of these points are now clarified in the main text. The general APE approach (Eq S4 of Supplement) works for any prior choice.

3. Figures: I found these hard to read. I realise that most people view on-line and therefore can greatly magnify figures, but a printed version is unreadable. In figure 7, I wonder if plotting Rt on a log-scale would help?

The figures have now been redone with better colour contrasts, larger labels and with fewer panels for clarity. Where appropriate (e.g. new Fig 10) Rt is now plotted on a log-scale.

4. The paper ends on a whimper, with it being unclear if the k=2 found from real data is true or an artefact of a noisy system. I’m surprised that the authors didn’t test the method against simulations that were made sequentially noisier, this would seem an obvious test.

To an extent the moving average filter we applied to the data was meant to do exactly that and did hint that k = 2 is indeed necessary for the Poisson model to best predict the data. However, we did not go into further detail as a separate study applying APE to negative binomial models, which can naturally account for some systematic heterogeneities in the data, is currently under development. Once complete the APE score of both negative binomial and Poisson models can be compared to settle this debate. We have now clarified our stance in the Discussion pointing out that k* = 2 is justified under the assumption of a Poisson model but that care also needs to be taken to assess the level of model mismatch.

I also feel that the speculation in the last paragraph of the results would sit far better in the discussion.

As requested, the last paragraph of the results has been removed and its content integrated within the Discussion.

**Reviewer: 2**

Renewal equation-based methods are frequently used to estimate the reproductive number ($R$) during infectious disease outbreaks. During an outbreak the $R$ varies with time, for instance due to susceptible depletion. Therefore, one challenge (and also use) for renewal-equation approaches is to estimate this variation. As the authors note, estimating variation in $R$ is typically accomplished using moving-window methods: dragging a window of a fixed size $k$ across the time series and estimating $R$ using data that fall within the window, assuming that $R$ is approximately constant within the window.

As a moving-window based method, this then poses the perennial problem of how to choose the window size $k$. To date, in the epidemiological literature this decision has typically been made on heuristic grounds. The contribution of this paper is to present a much-needed systematic way of making this decision, using techniques from information theory.

I consider the novel methodology presented a useful contribution to the disease modelling literature, and that the paper will ultimately be suitable for publication in PLoS Computational Biology.

Many thanks for the positive appraisal.

In the main, the methodology is explained clearly, in particular in the text, however I have few suggested comments that I feel would improve the paper and aid the reader in using the techniques presented. I do not think any of my suggestions will require the authors to perform any new analyses or generate any new data.

1. As the authors state in the introduction, they intend their method to be integrable “within existing real-time frameworks”. When reading the methods and the first part of the results (Figures 2-3) I was left unsure whether this was possible. The text gave me the impression that it was necessary to perform the analysis on the entire time series in order to use APE to find $k^{*}$. This would obviously make the method useless in an unfolding outbreak situation, where a decision would need to be made about choices of $k$ early on.

The authors later indirectly address this point in the section on “Diagnosing Efficacy of Control” and in Figure 4, which for me is actually the most important figure and section in the paper. I think its present framing in terms of control efficacy might obscure/undersell these results and its connection with the possibility for “integration within existing real-time frameworks”. I suggest the authors rework this section and expand the discussion of prospects for real-time implementation based on their findings. A few clarifying comments in the methods about how APE could be used in real-time would also help address this.

This section has now been reframed and reworded to better describe and clarify both the real-time applications and benefits of the APE method – its heading has also been updated to better reflect this emphasis. The main text now emphasises how APE can be used to assess, across an unfolding epidemic, the impact of recent interventions or to diagnose when controls should be elevated or to pinpoint when they can be relaxed.

2. For most of the examples the APE appears to select a minimal/maximal $k^{*}$: either 2 or $t/2$. Is this a general result, are there examples of the selection of intermediate values? It it illuminating to plot the APE curve as a function of $k$? e.g. is it a strictly monotonic function or does it possess bimodalities? This information is sort of contained in Figure 5 through plotting multiple lines for different values of $k$, but it is a little hard to interpret. Is $\text{APE}(k)$ flat at the change-times? Some explanation of these observations seems essential to me. For instance, one take-away from this paper for me is that, even if they don’t use APE, users of renewal-equation methods (e.g. EpiEstim) would be advised to pick both the minimal and maximal values of $k$, as one of them will be the best.

While a very interesting observation, it is unfortunately not a rule-of-thumb that can be suggested. The new Fig 2 and 3 show that intermediate values of k* are indeed possible. In the empirical examples a minimal k does seem to be required but we suspect this could be due to model-mismatch, and potentially indicative of the need to account for overdispersion. To perhaps emphasize the point, we include here some APE results from an ongoing analysis of COVID-19 in Italy (which will form part of a future publication – the initially high Rt is due to sparse data). Observe that k* = 8 is chosen out of values ranging from 2-22. The APE curve is also provided.

3. Figure 1 is very dense, and I found challenging to follow the algorithm. For instance, I took away from the figure that $k_{1}$ and $k_{2}$ were calculated at different time points ($t_{1}$ and $t_{2}$), using different data — whereas they are calculated using moving windows. I wonder if an annotated simulated example with multiple panels (e.g. simulated time series in panel a, panel b and c shows estimates of $R$ using $k_{1}$ and $k_{2}$ respectively, then the APE algorithm in panel d) might frame the problem and illustrate the algorithm better.

Thanks for the suggestion. Fig 1 has now been converted into a multi-panel form with a step-by-step flow of the algorithm presented. Hopefully it is now more intelligible, and the additional annotations help clarify the APE method.

4. Due to the number of different simulated scenarios analysed in the paper (6), it is difficult to comprehend Figure 2 and extract the key pieces of information. I also found the cyan confidence intervals a little hard to make out on a white background and some of the axis labels too small to read without zooming in. Is it possible to relegate some of the examples to the supplemental material and focus on one or two important ones in Figure 2?

The number of panels have now been reduced (see earlier responses).

Minor comments:

1. At the start of the methods it states, “incidence or number of infected cases in an epidemic at present time t”. The wording of this is slightly ambiguous, as “number of infected cases in an epidemic at present time t” suggests the total number of infectious individuals present in the population rather than the number of new cases (i.e. incidence) studied in the paper.

Thanks, this ambiguity has now been rectified. It is indeed the number of new cases.

2. Please add left and right y-axis labels to Figure 3 and 6.

Done or text has been included on the Figs to specify these labels.

3. Please add left y-axis labels to Figure 5.

Done.
